# Ontology-guided machine learning outperforms zero-shot foundation models for cardiac ultrasound text reports

**DOI:** 10.1038/s41598-024-83540-y

**Published:** 2025-02-14

**Authors:** Suganya Subramaniam, Sara Rizvi, Ramya Ramesh, Vibhor Sehgal, Brinda Gurusamy, Hikmatullah Arif, Jeffrey Tran, Ritu Thamman, Emeka C Anyanwu, Ronald Mastouri, G. Burkhard Mackensen, Rima Arnaout

**Affiliations:** 1 University of California, San Francisco, 521 Parnassus Avenue Rm 6222, San Francisco, CA 94143 USA; 2 University of California, Berkeley, Berkeley, CA USA; 3 University of Washington, Seattle, WA USA; 4 University of Arizona, Tucson, AZ USA; 5 University of Pittsburgh, Pittsburgh, PA USA; 6 University of Pennsylvania, Philadelphia, PA USA; 7 Indiana University, Indianapolis, IN USA

**Keywords:** Natural language processing, Machine learning, Large language models, Echocardiography report, Ontology, Cardiology, Medical imaging, Machine learning

## Abstract

Big data can revolutionize research and quality improvement for cardiac ultrasound. Text reports are a critical part of such analyses. Cardiac ultrasound reports include structured and free text and vary across institutions, hampering attempts to mine text for useful insights. Natural language processing (NLP) can help and includes both statistical- and large language model based techniques. We tested whether we could use NLP to map cardiac ultrasound text to a three-level hierarchical ontology. We used statistical machine learning (EchoMap) and zero-shot inference using GPT. We tested eight datasets from 24 different institutions and compared both methods against clinician-scored ground truth. Despite all adhering to clinical guidelines, institutions differed in their structured reporting. EchoMap performed best with validation accuracy of 98% for the first ontology level, 93% for first and second levels, and 79% for all three. EchoMap retained performance across external test datasets and could extrapolate to examples not included in training. EchoMap’s accuracy was comparable to zero-shot GPT at the first level of the ontology and outperformed GPT at second and third levels. We show that statistical machine learning can map text to structured ontology and may be especially useful for small, specialized text datasets.

## Introduction

Big data has the potential to revolutionize cardiac ultrasound (echocardiography) by enabling novel research and rigorous, scalable quality improvement^1^. Text reports are a key component of such analyses, serving as the prime means of communication for imaging findings^2^ and as a source of data labels for machine learning (ML) research.

Currently, cardiac ultrasound reports include both structured and free text and vary across institutions, hampering attempts to mine text for useful insights. Alternatively, mapping report text to a standardized ontology can help harmonize reports across institutions, languages, and imaging modalities^3^.

Several medical ontologies exist^4–7^ and with them, tools for entity extraction and linkage. For example, the Unified Medical Language System (UMLS)^4^ is supported in some NLP software packages (e.g. scispacy, pymetamap). However, UMLS is focused on describing terms found in clinical notes rather than the structure and attribute details important in cardiac ultrasound reporting, as we will demonstrate below. Radlex^6^, developed and maintained by the Radiological Society of North America (RSNA), is not as well supported by python but contains additional attributes, for example on patient status and study protocol, that may be useful for cardiac ultrasound as well. Furthermore, researchers have used machine learning to develop cross-lingual mappings to Radlex^8^. To date, however, neither cardiac ultrasound nor radiology report text is routinely mapped to an ontology in clinical practice.

With respect to cardiac ultrasound text reports, many published methods for extraction of numbers or other entities use tailored regular expressions and rule- or pattern-based algorithms^9–12^. These approaches are an effective use of small datasets, but they can require considerable effort to develop and can be poorly generalizable to additional concepts or institutions outside the training set. Most did not test externally^9–12^, and one that did showed recall near 50% on the external test set^13^.

Machine learning has been used on clinical notes, including some imaging text reports, but not specifically for extraction of concepts from cardiac ultrasound reports. Many have used various implementations of BERT (Bidirectional Encoder Representations from Transformers), an early LLM, to extract radiographic clinical findings^14^, mentions of devices^15^, study characteristics^16^, and result keywords^17^ from radiology or pathology reports. More recently, larger LLMs such as GPT have been used, for example to extract symptoms like “sore throat” and “cough” from text^18^. However, challenges to date in analyzing reports with LLMs include small corpus size, domain-specific language, and high need for accuracy. Performance of these models is therefore often lower than what would be required clinically without additional feature engineering^14,16^ or fine-tuning on thousands of manually-derived labels^15,17^ specific to the task. This likely reflects the fact that medical report text has specific structure and meaning while comprising only a small proportion of the general language used to train these models. Newer, larger LLMs like the Generative Pre-trained Transformer (GPT) hold greater promise for zero-shot inference^19,20^ (making predictions without the need for transfer learning or fine-tuning) so that all curated data may be used for testing. To our knowledge these models have not been applied specifically to cardiac ultrasound report text.

In this work, we use NLP to extract all qualitative components of an adult transthoracic echocardiogram (TTE) cardiac ultrasound report and map them to a standardized hierarchical ontology in a way that accommodates both free and structured text from across institutions. We compare a statistical ML model we developed, EchoMap, against zero-shot inference using GPT.

## Results

An ontology was created from the UCSF data dictionary as a proof-of-concept ontology designed to capture most relevant TTE descriptors within three or fewer hierarchical levels (Table 1). We then tested the ability of (i) a hierarchical random forest (RF) statistical machine learning model, termed EchoMap, trained on a small corpus of cardiac ultrasound sentences and (ii) zero-shot inference from GPT to map cardiac ultrasound report sentences to the ontology. Overall, EchoMap outperformed GPT.

**Table 1:** Example sentences from TTE reports and representative ontology mappings.

	In TTE ontology		Not in TTE ontology
	In UMLS/Radlex	Not in UMLS/Radlex	
Example Sentence	LV systolic function appears hyperdynamic	Small VSD is seen in the perimembranous septum	There is no aortic valve mass present
L1	Left ventricle	Interventricular septum	Aortic valve
L2	Function	Defect	Mass
L3	Hyperdynamic	Small perimembranous	None

### Structured reporting text varies across institutions, and existing medical ontologies do not contain all terms relevant to echocardiography

Despite all adhering to clinical guidelines, there were notable differences by institution in what structural and functional information was included in structured reporting. Our proof-of-concept TTE ontology (that captures all concepts in the UCSF data dictionary) captured only 57–68% of concepts in the data dictionaries from the other institutions in our test set (Table 2).

**Table 2:** Sentences in external data dictionaries represented by TTE ontology.

Data sets	Sentences represented in TTE ontology
UCSF validation (n = 228)	228 (100%)
UCSF free text (n = 1178)	914 (77.6%)
UCSF outside hospital (n = 338)	260 (76.9%)
UPITT (n = 777)	485 (62.4%)
IU (n = 906)	585 (64.6%)
UAZ (n = 825)	557 (67.5%)
UPENN (n = 1558)	891 (57.2%)
UW (n = 445)	259 (58.2%)

Additionally, mainstream ontologies like UMLS and Radlex contained some, but not all, of the echo-specific terms from the TTE ontology (Table 3). At each level of our ontology, UMLS contained a higher proportion of terms than Radlex did (while Radlex includes additional terms describing radiology study quality and protocol, those are not part of the current TTE ontology). Level 1 of the ontology, containing more common cardiac structures, was best covered in both UMLS and Radlex, followed by Level 2, and then Level 3, which each contain structures and observations successively more specific to cardiac ultrasound findings.

**Table 3:** TTE ontology concepts represented in existing ontologies.

	UMLS	Radlex
Overall (n = 260)	189 (72.7%)	84 (32%)
TTE ontology level		
Level 1 (n = 27)	24 (89%)	18 (67%)
Level 2 (n = 102)	88 (86%)	39 (38%)
Level 3 (n = 150)	95 (63%)	39 (26%)

### Machine learning can map echocardiography report text to an ontology

**Fig. 1:**
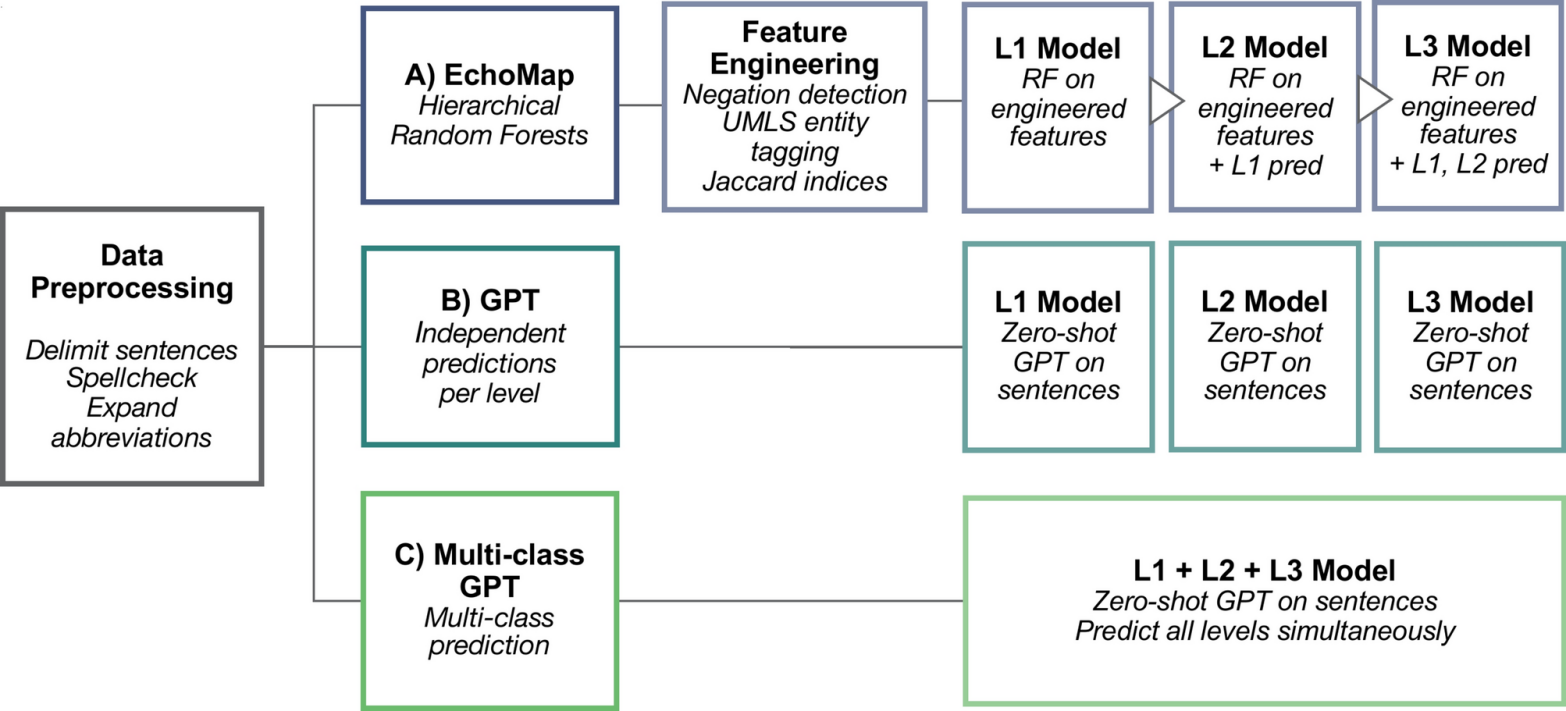
Workflow for the three machine learning approaches evaluated. Data (structured dictionaries and free text from echo reports) were preprocessed, then passed to each of three model types: (**A**) Hierarchical random forest statistical machine learning model, which included additional engineered features and used each level’s prediction to inform the subsequent level, (**B**) zero-shot GPT making independent predictions per level of ontology, (**C**) zero-shot GPT making multi-class prediction. GPT, generative pre-trained transformer; RF, random forest; UMLS, unified medical language system; L1, L2, L3, Level 1, Level 2, Level 3, respectively.

We tested two machine learning models for mapping cardiac ultrasound report sentences to our TTE ontology. The first was EchoMap, a statistical machine learning model trained on a portion of the UCSF data dictionary (see Methods). The second was single-shot inference using GPT. We used GPT in two ways: (i) single-shot inference to predict all three ontology terms at once (multi-class inference), and (ii) single-shot inference to predict Level 1, Level 2, and Level 3 terms separately (Fig. 1).

EchoMap is a three-level, hierarchical RF model that made use of engineered features including negation detection, UMLS named entity recognition, and Jaccard indices on sentence trigrams (Methods, Fig. 1). Additionally, for the L2 and L3 RFs, the result of the previous level’s RF was added as a feature.

For EchoMap, the balance of the UCSF dictionary served as a validation dataset, and seven additional datasets served as test datasets. These included the data dictionaries from the University of Washington, the University of Pennsylvania, the University of Pittsburgh, Indiana University, and the University of Arizona, as well as free text sentences from UCSF reports, and report sentences from a group of 18 outside hospitals incidentally found in the UCSF database. Because GPT was used “out of the box” rather than fine-tuned on any of our cardiac ultrasound text, the UCSF data dictionary also served as a test set with respect to GPT.

**Fig. 2:**
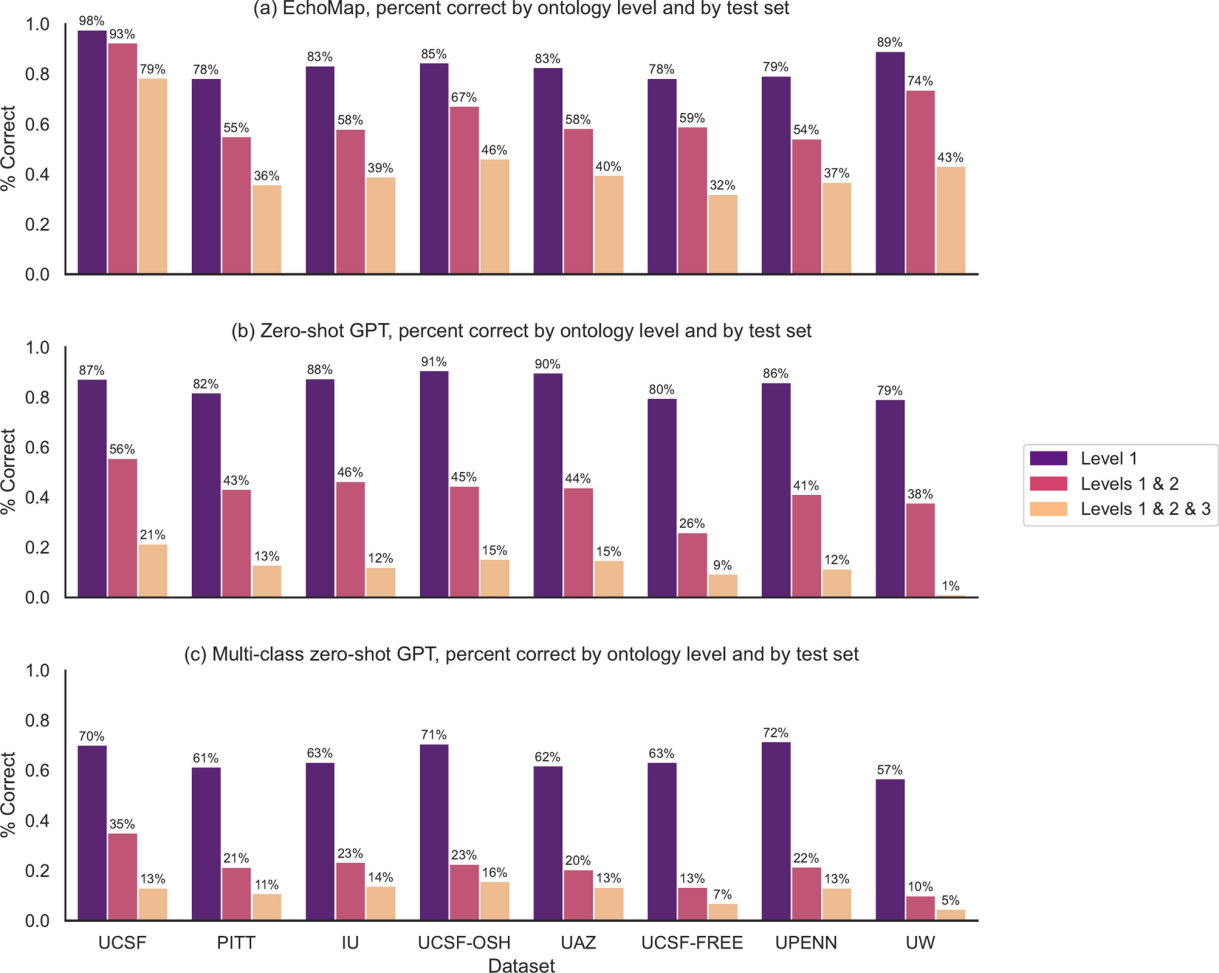
Correctness by ontology level, by dataset. Validation (UCSF) and test set (outside hospitals) performance for each of three model architectures: (**A**) Echomap, (**B**) Zero-shot GPT, (**C**) multi-class zero-shot GPT. UCSF, University of California, San Francisco. PITT, University of Pittsburgh; IU, Indiana University; UCSF-OSH, outside hospital reports in the UCSF system; UAZ, University of Arizona; UCSF-FREE, free-text sentences from UCSF reports; UPENN, University of Pennsylvania; UW, University of Washington.

EchoMap’s validation accuracy was 98% for the first level of the ontology, 93% for the first and second levels together, and 79% for the first, second, and third levels (Fig. 2A). Notably, Level 1 contained the fewest different ontological terms (n = 27) with the most representation in UMLS and was therefore the least difficult task. Levels 2 and 3 were more complex (containing 102 and 150 distinct terms, respectively) and contained less representation in UMLS, as mentioned above (Table 3).

EchoMap retained good first-level performance across test datasets, with a mean of 82 ± 4% correct on Level 1 (range 78–89%), 61 ± 7% cumulatively on Levels 1 and 2(range 54–74%), and 39 ± 5% cumulatively on all three levels (range 32–46%, Fig. 2A). Level 1 performance on the test datasets was lower than performance on the validation dataset (one-sample t-test *p* < 0.001). For Levels 1 and 2 together, and for all three levels together, test dataset performance was also statistically significantly different from performance on the validation dataset (*p* < 0.001, *p* < 0.001 respectively).

### A small, statistical machine learning model was superior to zero-shot and few-shot inference from a large language model

Given the rapidly improving capabilities of newer LLMs for myriad language tasks, we also tested the ability of GPT to map cardiac ultrasound text to the ontology. Using zero-shot inference allowed us to leverage the power of GPT without sacrificing any of the small dataset for training or fine-tuning; zero-shot inference has recently been shown to meet or beat fine-tuned performance at some medical tasks^21^.

GPT was used in two different ways. First, level 1, level 2, and level 3 were each predicted independently for each test sentence. Second, all three levels were predicted simultaneously for each sentence.

Using the independent classification approach, GPT gave 85 ± 5% correct for Level 1 (range 79–91%), 41 ± 7% correct for Levels 1 and 2 (range 26–46%), and 11 ± 5% correct for all three levels (range 1–15%). Using simultaneous classification for all three levels, performance was 64 ± 5% for Level 1 (range 57–72%), 19 ± 5% for Levels 1 and 2 (range 10–23%), and 11 ± 4% for all three levels (range 5–16%). These results are shown in Fig. 2.

**Fig. 3:**
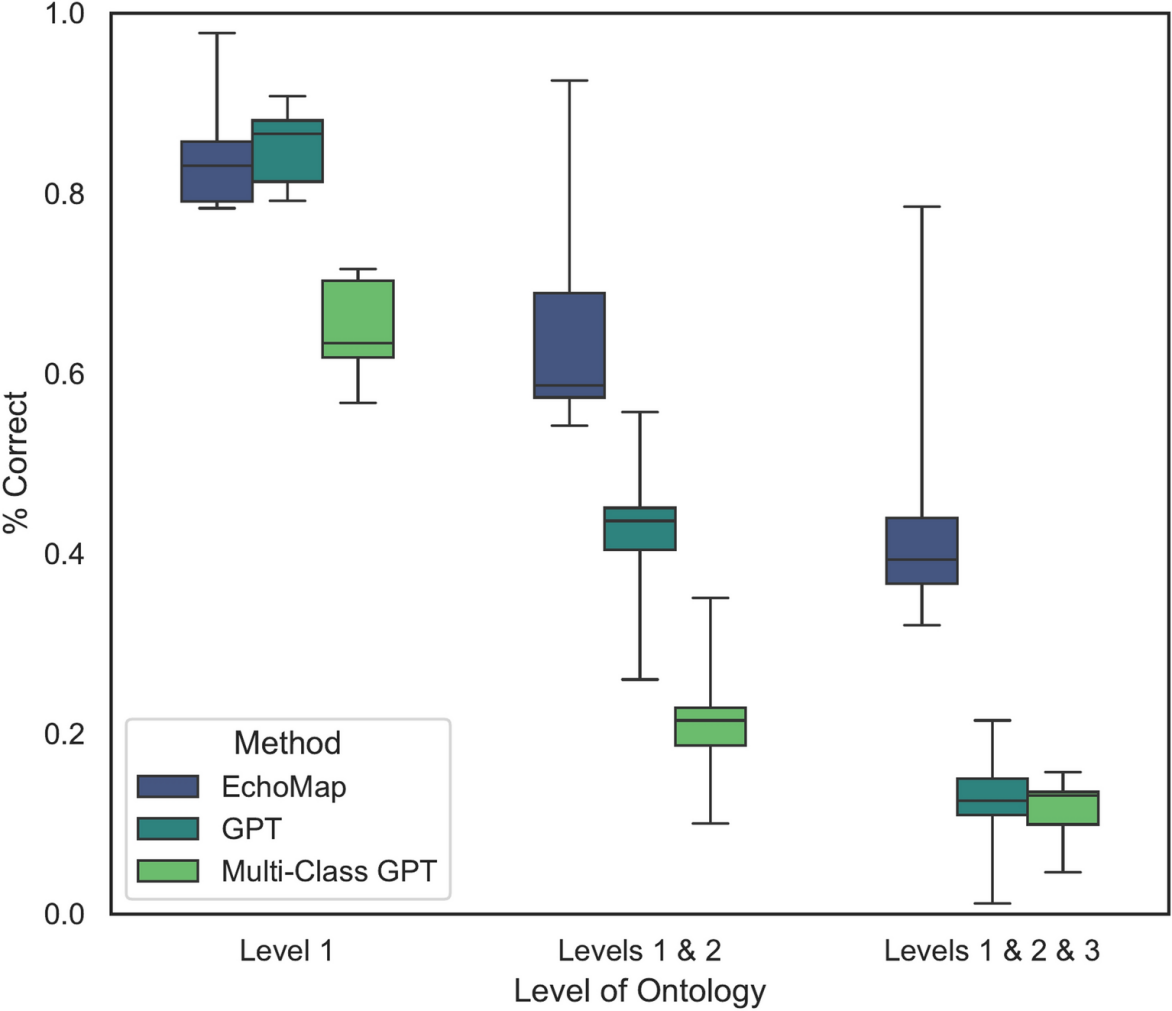
Aggregate performance across all datasets evaluated, by each mapping model and by ontology level. Box plots represent performance of all eight validation and test datasets in order to illustrate differences among mapping models.

We compared test performance across the three methods: EchoMap, GPT with individual classifications, and multi-class GPT (Fig. 1). EchoMap was statistically superior to GPT at all three ontology Levels (Friedman’s Χ^2^ *p* < 0.01 at Level 1, *p* < 0.001 at Levels 1 and 2, and *p* < 0.01 at Levels 1, 2, and 3) (Fig. 3). Only at Level 1, independent classification GPT, but not multi-class GPT, was statistically similar to EchoMap (pairwise *p* = 0.6 for individual GPT vs. EchoMap; *p* < 0.05 for multi-class GPT vs. EchoMap). At Levels 1, 2, and 3 together, both methods for GPT inference performed poorly and were statistically indistinguishable from each other (pairwise *p* = 0.9). Few-shot GPT fared similarly to zero-shot (Supplemental Table 4).

### EchoMap performance relies most on UMLS entities and simple text overlap

In the development of the EchoMap statistical ML model, different engineered features were tested (see Methods). In addition to negation, Jaccard similarities, UMLS named entity recognition, and hierarchical predictions, all used in the final EchoMap model, position of speech embedding as well as BioWordVec embedding were also evaluated against the validation dataset for Level 1, Level 2, and Level 3 of the ontology (Supplemental Table 3).

Simple Jaccard indices between sentences and ontological terms worked well. Overall, UMLS named entity recognition was the most useful embedding compared to position of speech or BioWordVec. UMLS embeddings were more useful for L1 performance than for L2 or L3, likely consistent with the fact that fewer ontological terms from L2/L3 are currently represented in UMLS (Table 3).

We investigated the impact of including hierarchical prediction (i.e., including the Level 1 prediction in the L2 classifier, and including the Level 1 and Level 2 predictions in the Level 3 classifier) (Supplemental Table 3). A version of EchoMap without hierarchical prediction showed no effect on Level 1 (expected) or Level 2 prediction performance but resulted in a 2% decrease in Level 3 performance.

We also investigated the impact of the negation detection feature on model performance. As in the Methods, negation detection was performed using the negspacy Python package. Eight percent of sentences carried negation, and negspacy demonstrated 94% accuracy in flagging negation. In the ontology, negative terms (e.g. “None” or “Not Seen”) are represented in Level 2 (2.8% of sentences) and in Level 3 (5.5% of sentences; twice as common as in Level 2). Consistent with these observations, a comparison of model versions with and without a negation feature resulted in minor differences (1–2% performance) in L2 and L3 only (Supplemental Table 3).

Finally, we noticed that many sentences wrongly flagged by negspacy were instances where the words “normal” or “non” (as in “non-coronary cusp”) were present. We therefore re-ran the EchoMap model, but with manually annotated negation (Supplemental Table 3). Interestingly, L3 showed worse performance (79% vs. 83%) when ground-truth negation was used.

### ML models displayed the ability to extrapolate to ontological combinations not initially included in training

Across all 7 external test sets, there were 2076 sentences where each level of Level 1, Level 2, and Level 3 were able to be mapped using a rearrangement of existing ontology terms, even though that particular L1–L2–L3 combination was not present in the TTE ontology (Supplemental Table 1). EchoMap demonstrated an ability to map sentences not originally included in the training, mapping 359 of these sentences correctly. Multi-class GPT performed similarly, correctly mapping 354 of these sentences, while GPT making independent predictions per level performed worse, only mapping 128 correctly.

## Discussion

Harmonizing cardiac ultrasound report text is critical to leverage it for cross-institutional big-data research. In this study, we provide proof of concept for how to apply NLP to harmonize cardiac ultrasound report text.

Importantly, the models in this study do not just extract certain information from text but seek to map all echo report text to an ontology—a framework for knowledge. An ontology is key because, as Meta Chief AI Scientist Yann LeCun says, “there is a limit to how smart [language models] can be and how accurate they can be because they have no experience of the real world, which is really the underlying reality of language.”^22^ The finding that eight different accredited hospitals operating according to echocardiography guidelines have such different data dictionaries is important in and of itself and demonstrates the need for greater harmonization.

One reason echocardiography report text has not been mapped to an ontology to date is the technical challenges inherent in doing so—challenges we address in this proof-of-concept study. Regular expressions-based text extraction is tedious and brittle, while the corpus of echo text is so small that machine learning is hard to train or transfer-learn^17^—especially for those large language models that seem to excel at more general language tasks. Indeed, a key challenge in this use case is the small size of structured echo report sentences, which makes any trained ML model (whether trained from scratch or fine-tuned) run the risk of overfitting, yet such data-poor, high-stakes use cases abound in medical text. We show that a small statistical ML model, EchoMap, trained on a small amount of the echo report corpus can actually outperform zero-shot inference from prevailing LLMs.

With EchoMap, we see a relationship between each level’s performance and the number of ontology terms present in UMLS; longstanding ontologies that have proved so powerful for analysis of clinical notes are not optimized for cardiac ultrasound text reports. This suggests that further investment of adding echo-specific terms to UMLS (or alternatively, Radlex with greater software support for feature embeddings) could pay dividends both in improving EchoMap and in achieving greater representation of echocardiography concepts in more general medical ontologies.

Sub-analysis to investigate the impact of hierarchical features and negation used in EchoMap were similarly revealing. Hierarchical features had little impact on Level 2 prediction performance, possibly because the ontology already includes one-to-many relationships between Level 1 and Level 2 terms (e.g., “regurgitation” in Level 2 can legitimately refer to an Level 1 of either “aortic valve, “mitral valve,” “pulmonic valve,” or “tricuspid valve.”). Hierarchical features had a greater impact on Level 3 prediction performance, although still modest (2%). Level 3 has a larger number of classes and therefore appears to benefit from the additional constraints provided by the Level 1–Level 2 prediction features. Overall, these data suggest that a one-off misclassification at one level of the ontology would have only minor consequences for overall performance.

It is interesting to note that EchoMap performed worse in Level 3 prediction when ground-truth features for negation were used instead of negspacy. We believe this is because negspacy’s errors were not random—it flagged “normal” and “non”—and that this information, while not a correct labeling of negation, was nevertheless a useful feature for the random forest models in predicting those ontology terms. While this behavior was not expected, the explainable nature of random forest models proved an advantage over large language models in detecting this issue.

There are several limitations of the current study, which we see as areas for future improvement. A major limitation is that the cardiac ultrasound ontology must be improved and expanded. The ontology used in the current study was created from only one institution’s data dictionary (along with clinical domain knowledge), so that other institutions’ dictionaries could be reserved for testing. An improved ontology could start with more data dictionaries and could expand to all areas germane to echo reporting, including study quality, patient status, pediatric congenital heart disease, stress and transesophageal echocardiography, and more. With more institutional sharing of data dictionaries, the ontology can be improved.

Second, despite testing of several versions of EchoMap and two versions of GPT, more ML model exploration and improvement may further improve performance and generalizability. While a small, hierarchical random forest model was chosen in order to leverage our small training dataset, slightly worse results on the external hospital datasets suggest that overfitting may still be an issue that will need to be improved upon in future work. LLM fine-tuning and other approaches can be considered in the future. Additionally, future work will benefit from more deft handling of negation, compound sentences and other complexities.

In conclusion, mapping echocardiographic report text to a standardized ontology can aid in data mining efforts for both quality improvement and machine learning research. While LLMs have risen in popularity, small statistical machine learning models also perform well and may be especially useful for small, specialized text datasets where clinical meaning is important. These results highlight the utility of continuing to develop an open-source, high-resolution, standardized cardiac ontology to harmonize reports across institutions.

One can envision a future where an optimized ontology is developed and maintained by the echocardiography community, with investment into depositing all terms into UMLS or similar general medical ontologies, and where all institutions can map their data dictionaries to this central and comprehensive resource.

## Materials and methods

### Ontology construction

We developed a three-tier ontology for the echocardiographic anatomic structures, functional elements, and descriptive characteristics in adult transthoracic echocardiograms. UCSF’s data dictionary (standard phrases that populate structured reporting, n = 951 sentences) was used as a starting point. This starting point was then evaluated against clinical guidelines and other institutions’ data dictionaries for completeness^23^, and structures or attributes not mentioned in UCSF dictionaries were added. Then all terms were mapped to UMLS (https://uts.nlm.nih.gov/uts/umls/home) and RadLex (https://radlex.org/) identifiers where available. The resulting ontology was then evaluated against expert knowledge for accuracy, conciseness, and consistency^23^ and any redundancies or contradictions were corrected. The final ontology included 260 distinct ontology terms across all three levels (five terms are found in both Level 2 and Level 3 of the ontology). Table 1 provides an example.

### Datasets

#### Training and validation

The UCSF data dictionary (n = 951 sentences) was split into a training set (n = 723 sentences) and separate validation set (n = 228 sentences).

#### Testing

Data dictionaries from the University of Arizona (n = 1202 sentences), Indiana University (n = 2143 sentences), University of Washington (n = 504 sentences), University of Pennsylvania (n = 2024 sentences), and University of Pittsburgh (n = 966 sentences) medical centers were used as five external test sets.

Two additional test sets were derived from patient cardiac ultrasound reports. All data was accessed and analyzed in accordance with the UCSF IRB. The experimental protocols were approved by the UCSF IRB. Informed consent is waived by the UCSF IRB. First, all patient echo reports from 1995 to 2021 were obtained from UCSF in accordance with the UCSF IRB and de-identified. 102 reports in the UCSF system had come from eighteen outside hospitals and were used for an “outside hospital” dataset (n = 483 unique sentences). Second, UCSF patient reports had data dictionary sentences removed so that only free-text sentences were remaining (n > 250,000 sentences). A random sample of these free-text sentences (1500 minus 32 sentence fragments removed, n = 1468 unique sentences) became the UCSF free-text dataset.

All training and test datasets were labeled with ontology terms by clinicians, to serve as ground truth.

### Text processing

Sentences were delimited, spell-corrected, abbreviations were expanded, and made lowercase.

### Feature engineering

For input into the statistical ML model, feature engineering was performed on each sentence in a four-step process as follows. Step 1: For each sentence, negation detection (https://pypi.org/project/negspacy/) was performed to tag sentences with negation absent versus present (0 vs. 1). Step 2: UMLS entities were extracted from each sentence (https://pypi.org/project/scispacy/ named entity recognition (NER), and principal component analysis (PCA) (https://scikit-learn.org/) was used to reduce this result into a 25-element vector. Step 3: Jaccard indices (a measure of overlap) were calculated between sentence trigrams and each ontology term. Step 4: For Levels 2 and 3, the classification of the previous model was included as a feature, such that the Level 1 classification was fed into the Level 2 RF, and the Level 2 classification was fed into the Level 3. RF. Steps 1–4 were concatenated into a single input vector. The final input to the Level 1 statistical model was 53 features (negation + 25-element UMLS + 27 Level 1 Jaccard indices). The final input to the Level 2 statistical model was 284 features (negation + UMLS + 27 Level 1 Jaccard + 230 Levels 2 and 3 Jaccards + Level1 prediction). The final input to the Level 3 statistical model was 285 features (Level 2 features + Level 2 prediction). Supplemental Table 2 summarizes the features used for each level.

Additional features calculated, but not used in the final model, include a 25-element embedding from position of speech tagging (“en_core_web_sm” model from scispacy, then reduced to 25 elements with PCA) and a 25-element embedding from the BioWordVec large language model^24^ (“BioWordVec_PubMed_MIMICIII_d200.vec.bin” from scispacy, reduced with PCA (Supplemental Table 3).

For input into GPT3.5, raw sentences were used without feature engineering.

### Model architectures, training, and inference

For the statistical ML model, random forest (RF) classifiers were used (https://scikit-learn.org/) for classification at each of the three ontology levels. GridSearch was used to help determine optimal hyperparameters for each classifier. The first RF classifier had a maximum depth of 25 and 100 estimators. The second classifier had max depth 40, number of estimators 120. The third classifier had max depth 25, number of estimators 100. All classifiers used the class balance option to mitigate class imbalance among different ontology terms.

For the large language model (LLM) classifier, OpenAI’s GPT 3.5 (a pre-trained model) was accessed via scikit-llm Python package (https://pypi.org/project/scikit-llm/; the zero-shot GPT classifier and multi-class zero-shot GPT classifiers were used, respectively, for one classification at a time vs. multi-class predictions). The few-shot versions of these classifiers were also used; for these experiments, the same training set used for the hierarchical RF classifier was used for the few-shot adaptation.

### Statistical analysis

Model predictions were compared to ground-truth labels, and percent correct was calculated both by ontology level and overall. One-sample t-test, Friedman Χ^2^, and Nemenyi post-hoc comparisons were used to perform statistical comparison across groups.

## Supplementary Information

Supplementary Information.

## Data Availability

Patient report data cannot be made available. The UCSF data dictionary can be made available upon reasonable request for non-commercial use and with approval; contact corresponding author.

## Code availability

Code will be made available at github.com/ArnaoutLabUCSF/CardioML/ upon publication.
