# Supplementary material for: Ontology-guided machine learning outperforms zero-shot foundation models for cardiac ultrasound text reports
Source: Sci Rep. 2025 Feb 14;15:5456. doi: 10.1038/s41598-024-83540-y (PMC11828978; doi:10.1038/s41598-024-83540-y)
Supplement: Supplementary file 1 — Supplementary Information. [file 41598_2024_83540_MOESM1_ESM.docx]

Supplemental Information

Ontology-guided machine learning outperforms one-shot foundation models for cardiac ultrasound text reports

Suganya Subramaniam, Sara Rizvi, Ramya Ramesh Vibhor Sehgal, Brinda Gurusamy, Hikamtullah Arif, Jeffrey Tran, Ritu Thamman, Emeka Anyanwu, Ronald Mastouri, G. Burkhard Mackensen, Rima Arnaout

## **Supplemental Table 1. Extrapolation performance on term combinations outside TTE ontology.**

|  | **Echomap** | **GPT** | **GPT Multi-Class** |
| --- | --- | --- | --- |
| **UCSF (n=0)** | n/a |  |  |
| **PITT (n=292)** |  |  |  |
| L1 | 237 (81%) | 260 (89%) | 199 (68%) |
| L1, L2 | 118 (40%) | 184 (63%) | 91 (31%) |
| L1, L2, L3 | 32 (11%) | 9 (3%) | 64 (22%) |
| **IU (n=321)** |  |  |  |
| L1 | 233 (73%) | 266 (83%) | 215 (67%) |
| L1, L2 | 137 (43%) | 186 (58%) | 71 (22%) |
| L1, L2, L3 | 46 (14%) | 22 (7%) | 45 (14%) |
| **UCSF-OSH (n=78)** |  |  |  |
| L1 | 41 (53%) | 61 (78%) | 38 (49%) |
| L1, L2 | 25 (32%) | 34 (44%) | 15 (19%) |
| L1, L2, L3 | 8 (10%) | 6 (8%) | 9 (12%) |
| **UAZ (n=268)** |  |  |  |
| L1 | 176 (66%) | 222 (83%) | 188 (70%) |
| L1, L2 | 90 (34%) | 131 (49%) | 62 (23%) |
| L1, L2, L3 | 46 (17%) | 35 (13%) | 46 (17%) |
| **UCSF-FREE (n=264)** |  |  |  |
| L1 | 152 (58%) | 177 (67%) | 137 (52%) |
| L1, L2 | 87 (33%) | 87 (33%) | 34 (13%) |
| L1, L2, L3 | 35 (13%) | 16 (6%) | 21 (8%) |
| **UPENN (n=667)** |  |  |  |
| L1 | 554 (83%) | 607 (91%) | 500 (75%) |
| L1, L2 | 272 (41%) | 407 (61%) | 267 (40%) |
| L1, L2, L3 | 149 (22%) | 40 (6%) | 160 (24%) |
| **UW (n=186)** |  |  |  |
| L1 | 136 (73%) | 112 (60%) | 82 (44%) |
| L1, L2 | 92 (49%) | 52 (28%) | 22 (12%) |
| L1, L2, L3 | 43 (23%) | 0 (0%) | 9 (5%) |

## **Supplemental Table 2. Summary of features input into each level of the EchoMap random forest classifiers.** The number of features for negation detection, UMLS embedding, Jaccard indices, and prior model prediction are shown, resulting in the total number of features in the input vector for each Level’s random forest model.

| **RF** | **Negation detection** | **UMLS embedding  (25 PCs)** | **Jaccard scores  (for each ontology term in that Level)** | **Previous level’s prediction** | **Total No. Features** |
| --- | --- | --- | --- | --- | --- |
| **Level 1** | 1 | 25 | 27 | N/A | 53 |
| **Level 2** | 1 | 25 | 257 | 1  (L1 prediction) | 284 |
| **Level 3** | 1 | 25 | 257 | 2  (L1 & L2 predictions) | 285 |

RF, random forest. UMLS, Unified Medical Language System. PCs, principal components.

## **Supplemental Table 3. Iterations on statistical ML model features.** Bold indicates the highest performance at each level. EchoMap is represented as the final entry in the table.

|  | **Features used** | | | | | | **Validation performance, % correct** | | |
| --- | --- | --- | --- | --- | --- | --- | --- | --- | --- |
| **Model Brief Description** | **Neg** | **Jac** | **Hier-archy** | **POS (PCA)*** | **BWV (PCA)*** | **UMLS (PCA)*** | **L1** | **L2** | **L3** |
| No Jaccard used | X |  | X | X (25) | X (25) | X (25) | 90 | 87 | 57 |
| Only embedding used is UMLS; no Jaccard | X |  | X |  |  | X (25) | 91 | 83 | 54 |
| No embeddings used | X | X | X |  |  |  | 92 | 92 | 81 |
| Only embedding used is POS | X | X | X | X (25) |  |  | 95 | 90 | 80 |
| No UMLS embedding used | X | X | X | X (25) | X (25) |  | 95 | 92 | 81 |
| Only embedding used is BioWordVec | X | X | X |  | X (25) |  | 95 | 92 | 81 |
| Only embedding used is UMLS; only 10 PCA elements | X | X | X |  |  | X (10) | 96 | 90 | **83** |
| No negation used |  | X | X | X (25) | X (25) | X (25) | 97 | 91 | 81 |
| No POS embedding used | X | X | X |  | X (25) | X (25) | 97 | 91 | 81 |
| UMLS, Jaccard, and Hierarchical only |  | X | X |  |  | X (25) | 97 | 92 | 80 |
| UMLS, and Jaccard only |  | X |  |  |  | X (25) | 97 | 92 | 80 |
| All features | X | X | X | X (25) | X (25) | X (25) | 97 | **93** | 82 |
| No BioWordVec embedding used; no hierarchical pred | X | X |  | X (25) |  | X (25) | **98** | 92 | 79 |
| No BioWordVec embedding used | X | X | X | X (25) |  | X (25) | **98** | **93** | 81 |
| Only embedding used is UMLS; no hierarchical pred | X | X |  |  |  | X (25) | **98** | **93** | 81 |
| Only embedding used is UMLS (EchoMap) | X | X | X |  |  | X (25) | **98** | **93** | **83** |
| EchoMap, negation detection manually performed | X(GT) | X | X |  |  | X(25) | **98** | 92 | 79 |

*indicates the number of principal components used

PCA, principal components analysis. Neg, negation detection. Jac, Jaccard indices.

Hierarchy, Hierarchical predictions across levels. POS, position of speech tag embedding. BWV, BioWordVec embedding. UMLS, UMLS named entity recognition embedding.

GT, ground-truth label used

**Supplemental Table 4. Performance of few-shot GPT.**

| **DataSet** | **Few Shot GPT, % correct L1** | **Few Shot GPT, % correct L1L2** | **Few Shot GPT, % correct L1L2L3** | **Multi-class Few Shot GPT, % correct L1** | **Multi-class Few Shot GPT, % correct L1L2** | **Multi-class Few Shot GPT, % correct L1L2L3** |
| --- | --- | --- | --- | --- | --- | --- |
| **Val** | 0.79 | 0.58 | 0.23 | 0.77 | 0.59 | 0.26 |
| **PITT** | 0.64 | 0.28 | 0.08 | 0.64 | 0.31 | 0.13 |
| **IU** | 0.7 | 0.3 | 0.06 | 0.68 | 0.29 | 0.12 |
| **UCSF-OSH** | 0.72 | 0.35 | 0.1 | 0.75 | 0.45 | 0.19 |
| **UAZ** | 0.75 | 0.3 | 0.08 | 0.77 | 0.33 | 0.11 |
| **UCSF-FREE** | 0.75 | 0.34 | 0.07 | 0.73 | 0.35 | 0.09 |
| **UPENN** | 0.68 | 0.27 | 0.08 | 0.7 | 0.31 | 0.13 |
| **UW** | 0.7 | 0.39 | 0.09 | 0.73 | 0.4 | 0.13 |
